# Schistosome-Associated Pulmonary Arterial Hypertension: A Review Emphasizing Pathogenesis

**DOI:** 10.3389/fcvm.2021.724254

**Published:** 2021-10-05

**Authors:** Teresa Cristina Abreu Ferrari, Ana Cristina Lopes Albricker, Ina Morais Gonçalves, Cláudia Maria Vilas Freire

**Affiliations:** ^1^Departamento de Clínica Médica, Faculdade de Medicina, Universidade Federal de Minas Gerais, Belo Horizonte, Brazil; ^2^Hospital das Clínicas, Universidade Federal de Minas Gerais, Belo Horizonte, Brazil; ^3^Programa de Pós-Graduação em Ciências Aplicadas à Saúde do Adulto, Universidade Federal de Minas Gerais, Belo Horizonte, Brazil; ^4^Graduação em Medicina, Centro Universitário de Belo Horizonte, Belo Horizonte, Brazil

**Keywords:** pulmonary hypertension, pulmonary arterial hypertension, schistosomiasis, hepatosplenic schistosomiasis, TGF-β

## Abstract

Schistosomiasis, especially due to *Schistosoma mansoni*, is a well-recognized cause of pulmonary arterial hypertension (PAH). The high prevalence of this helminthiasis makes schistosome-related PAH (Sch-PAH) one of the most common causes of this disorder worldwide. The pathogenic mechanisms underlying Sch-PAH remain largely unknown. Available evidence suggests that schistosome eggs reach the lung via portocaval shunts formed as a consequence of portal hypertension due to hepatosplenic schistosomiasis. Once deposited into the lungs, the eggs elicit an immune response resulting in periovular granuloma formation. Immune mediators drive transforming growth factor-β (TGF-β) release, which gives rise to pulmonary vascular inflammation with subsequent remodeling and development of angiomatoid and plexiform lesions. These mechanisms elicited by the eggs seem to become autonomous and the vascular lesions progress independently of the antigen. Portopulmonary hypertension, which pathogenesis is still uncertain, may also play a role in the genesis of Sch-PAH. Recently, there have been substantial advances in the diagnosis and treatment of PAH, but it remains a difficult condition to recognize and manage, and patients still die prematurely from right-heart failure. Echocardiography is used for screening, and the formal diagnosis requires right-heart catheterization. The experience in treating Sch-PAH is largely limited to the phosphodiesterase type 5 inhibitors, with evidence suggesting that these vasodilators improve symptoms and may also improve survival. Considering the great deal of uncertainty about Sch-PAH pathogenesis, course, and treatment, the aim of this review is to summarize current knowledge on this condition emphasizing its pathogenesis.

## Introduction

Pulmonary hypertension (PH) is a hemodynamic and pathophysiologic condition that comprises heterogeneous disorders, leads to right-heart failure if untreated, and carries substantial morbidity and mortality (1–4). It is defined as a mean pulmonary arterial pressure exceeding 20 mmHg at rest as assessed by right-heart catheterization (5). According to the recent World Health Organization (WHO) classification, it is grouped into five categories: pulmonary arterial hypertension (PAH) (Group 1), left-heart-related (Group 2), lung-related (Group 3), chronic thromboembolic (Group 4), and miscellaneous (Group 5) PH (5). Schistosome-associated PH is classified as Group 1.

Pulmonary arterial hypertension is a chronically progressive condition that results from elevation in precapillary pulmonary artery pressure due to inflammation, vascular tone imbalance, and progressive remodeling of the pulmonary vasculature (4). Schistosomiasis is a well-recognized cause of PAH, which is estimated to occur in about 5–15% of patients with the severe hepatosplenic form of schistosomiasis, particularly due to *Schistosoma mansoni* (6, 7). Although PAH is an uncommon complication of schistosome infection, the high prevalence of this helminthiasis makes schistosome-associated PAH (Sch-PAH) one of the most common causes of this disorder worldwide (7).

Patients with Sch-PAH present clinical, laboratory, and hemodynamic profiles similar to those observed in PAH due to other etiologies (8). However, the pathogenic mechanisms underlying Sch-PAH remain largely unknown (9, 10), though available evidence suggests that egg deposition into the lung and consequent inflammatory response are key events in the genesis of these disorder. Over the last two decades, there have been substantial advances in the diagnosis and treatment of Sch-PAH, but it remains a difficult condition to recognize and manage, and patients still die prematurely from right-heart failure (11). In synthesis, there is a great deal of uncertainty about Sch-PAH pathogenesis, course, and treatment. Therefore, the aim of this mini-review is to summarize the current knowledge regarding Sch-PAH emphasizing its pathogenesis.

## Etiopathogenesis and Course of Schistosome Infection

Schistosomiasis is a neglected tropical parasitic disease caused by trematode flukes of the genus *Schistosoma*. These blood flukes use man and other mammals as definitive hosts, and aquatic and amphibian snails as intermediate hosts. *Schistosoma mansoni, S. haematobium*, and *S. japonicum* are the most common disease-causing species and the most widely distributed, whereas *S. guineensis, S. intercalatum*, and *S. mekongi* occur in a few geographical areas and are only of local importance (12–15). According to available estimates, schistosomiasis affects more than 230 million people in parts of the Middle East, South America, Southeast Asia and, particularly, in sub-Saharan Africa (14, 15). The infection is more common in rural areas, but it also occurs in the periphery of urban centers associated with poor sanitation.

The schistosome species differ from each other in several characteristics and these differences are important determinants of the clinical presentations of the infection. *Schistosoma haematobium* inhabits the pelvic plexuses and damages the urinary tract. The other species reside in the portal and mesenteric veins, and cause intestinal and liver disease (12–15).

**Figure 1:**
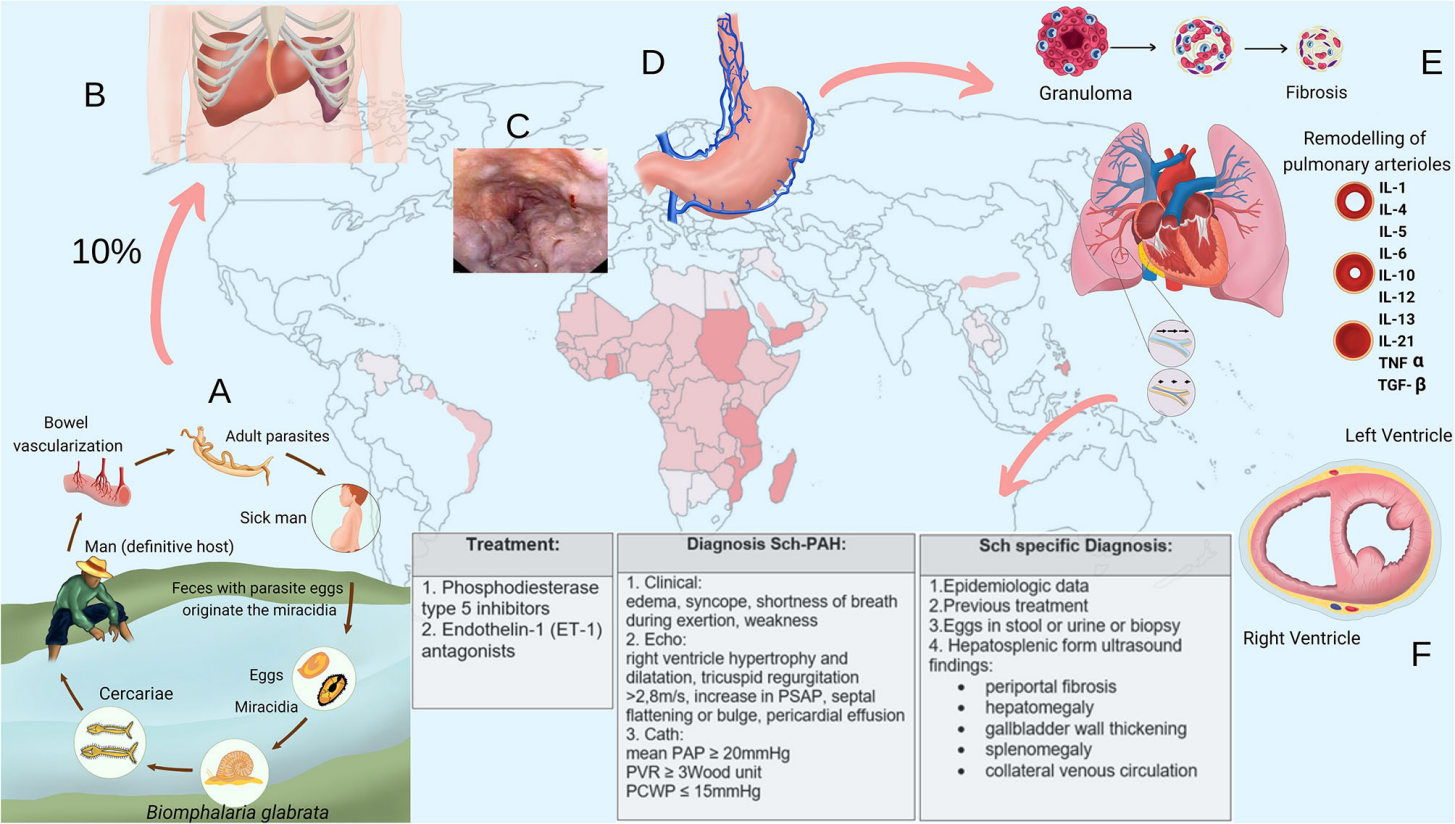
**(A)** Schistosome life cycle. **(B)** Enlarged liver and spleen in hepatosplenic schistosomiasis. **(C)** Endoscopic aspect of esophageal varices. **(D)** Collateral venous circulation. **(E)** Pulmonary vascular remodeling in schistosome-associated pulmonary arterial hypertension. **(F)** Right-heart strain (cross section of the heart). IL, interleukin; TNF, tumor necrosis factor; TGF, transforming growth factor; Echo, echocardiography; Cath, right-heart catheterization.

Schistosome eggs are shed into the environment through feces or urine. The eggs that reach freshwater release the ciliated miracidium larva that infects the intermediate host. After multiplying asexually into sporocysts and later into cercarial larvae, they are shed by the snail. On finding a definitive host, the cercariae penetrate the skin, lose their bifurcated tail transforming into schistosomula, which migrate in blood to the lungs and, then, to the liver, where they grow into adult worms, mate and follow the path to the mesenteric venules of the colon (*S. mansoni*), small intestine (*S. japonicum*), or pelvic plexus (*S. haematobium*), where eggs are laid (Figure 1A). About a third of the eggs are eliminated through feces or urine. Those not eliminated remain trapped in the intestinal or bladder wall, or are transported by blood to the liver or other organs (12–15). Eggs trapped into tissues elicit a cell-mediated periovular granulomatous reaction. As the infection progresses, a down modulation of the immune response originate progressive smaller granulomas (16), which are gradually replaced by fibrotic deposits (12, 15). Thus, in late infection, organ-specific clinical manifestations usually positively correlate with infection intensity, and are chiefly mediated by egg induced inflammation and granulomatous reaction (15, 16).

The course of schistosome infection is classified into acute and chronic phases and different clinical forms (17). Acute schistosomiasis is mostly seen in travelers after primary infection. Commonly, it presents as sudden onset of fever, malaise, myalgia, headache, eosinophilia, fatigue, diarrhea (with or without blood), abdominal pain, hepatomegaly, non-productive cough with pulmonary infiltrates on chest X-ray and, in case of *S. haematobium* infection, hematuria. These symptoms often subside spontaneously over a few weeks (15–17).

The chronic phase is usually asymptomatic, but individuals who live in endemic areas may present clinical manifestations that progress insidiously without specific treatment. The intensity and duration of the infection determine the severity of the chronic fibro-obstructive disease (12–17). In the intestinal form, eggs tapered into the gut wall provoke mucosal granulomatous inflammation that may cause chronic or intermittent abdominal pain and discomfort with or without diarrhea that may contain blood. Granulomatous inflammation around eggs embolized to presinusoidal periportal spaces of the liver that occur in the early stages of the chronic phase may cause hepatomegaly, which characterizes the hepatointestinal form. In long-standing intense infections, periportal collagen deposition leads to fibrosis and progressive occlusion of terminal branches of the portal veins causing portal hypertension. This clinical form is called hepatosplenic and presents with splenomegaly, collateral venous circulation, portocaval shunting, and esophagogastric varices [(12–14, 16, 17); Figures 1B–D]. The mild urinary form results from granulomatous inflammation around *S. haematobium* eggs tapered in the bladder and ureteral walls. Its defining symptom is hematuria, often associated with urinary frequency, burning micturition, dysuria and suprapubic discomfort. In high-intensity late chronic infections, obstruction of the urinary tract may develop due to accumulation of dead calcified eggs and fibrosis formation in vesical and ureteral walls (12–14, 16, 17).

Portosystemic shunts resulting from portal hypertension in hepatosplenic schistosomiasis enables egg embolization from the portal venous system to the systemic venous circulation, and then to the lungs, which may cause PH in about 5–10% of patients with this severe form of schistosomiasis, particularly when the etiologic agent is *S. mansoni* (7). Although preexisting hepatosplenic disease is considered essential for the development of Sch-PAH, in rare reported cases, it was described the occurrence of this condition in chronic schistosomiasis without evidence of portal hypertension (18). However, in these situations, it is not possible to rule out the possibility that the patients actually presented schistosome infection associated with PH of another etiology. This may also be the explanation for the rare reported cases of PH associated with *S. haematobium* infection (19).

## Schistosome-Related Pulmonary Arterial Hypertension

Schistosomiasis is the most common parasitic disease associated with PH (20, 21); and Sch-PAH might represent one of the most prevalent causes of this condition worldwide (22). Interest in this disorder has increased in recent years due to the current availability of PAH treatment. However, Sch-PAH is still underdiagnosed and undertreated.

Currently, Sch-PAH is defined by the combination of the following criteria: (1) mean pulmonary arterial pressure >20 mmHg, pulmonary arterial wedge pressure ≤15 mmHg (current criteria of precapillary PH) associated with a pulmonary vascular resistance >3 Wood Units, assessed at rest by right-heart catheterization; (2) history of schistosome infection, as evidenced by current or prior presence of schistosome eggs in stool examination or rectal biopsy, prior schistosome specific treatment, or prior exposure to the infection in a schistosome endemic area; and (3) ultrasonographic findings consistent with hepatosplenic schistosomiasis, which include periportal fibrosis, enlargement of the left lobe of the liver, and thickening of the gallbladder wall (23) associated with signs of portal hypertension such as splenomegaly and collateral venous circulation [(7); Figures 1B–D].

### Pathogenesis

The exact pathogenesis of Sch-PAH remains unclear; however, mechanical obstruction of lung vasculature by embolized eggs, pulmonary vascular inflammation and remodeling, and portopulmonary hypertension-like pathophysiology have been suggested as the most probably pathogenic mechanisms for this condition (6, 18). The immunopathogenesis of the disease is also unclear, although there are similarities with the immunologic features of idiopathic PAH (18).

After being embolized to lungs via portosystemic shunts, the eggs elicit a predominantly T helper type-2 (Th2) cells immune response resulting in periovular granuloma formation. These granulomas affect both lung parenchyma and pulmonary vasculature causing parenchyma damage and fibrosis, and a certain extend of distal pulmonary vascular bed loss with consequent elevation in pulmonary vascular resistance. This mechanism is similar to what occurs in liver after egg deposition in the portal branches of portal vein, which results in some grade of fibrosis, tissue destruction, and obstruction of the portal flow (7, 18). Although this mechanism was originally considered the principal explanation for Sch-PAH, currently, it has been suggested that mechanical obstruction of the pulmonary arteries by the eggs may not cause significant increase in pulmonary vascular resistance and that Sch-PAH is especially due to a proliferative vasculopathy [(18, 24); Figure 1E].

A diffuse and heterogeneously distributed pulmonary vasculopathy is an important pathogenic mechanism underlying Sch-PAH (7, 18, 24–26). Alterations in the structure and function of the endothelial cells develop in association with growth of neointimal, medial, and adventitial layers, resulting in an occlusive arteriopathy, which increases the resistance to the blood flow. Angiomatoid and plexiform lesions also develop (6, 7, 22, 26). The exact pathogenesis of this vasculopathy is still unclear, though inflammatory mechanisms are strongly suspected (27). Some evidence indicates that transforming growth factor (TGF)-β plays a key role in the vascular remodeling (7, 9, 28–30); and, in Sch-PAH, the release of this cytokine is probably a consequence of the Th2 inflammation elicited by eggs deposited into lungs through a series of cellular and signaling events (7, 9, 29). However, Sch-PAH shares TGF-β-dependent vascular remodeling with idiopathic, heritable and autoimmune-associated etiologies of PAH (30). An interesting aspect is the fact that TGF-β activation, seems to become autonomous and independent of schistosome antigen resulting in a persistent vascular disease despite parasite eradication (7, 31). Some evidence suggests that TGF-β can induce a shift from glucose oxidation toward uncoupled aerobic glycolysis in pulmonary artery smooth muscle cells (PASMCs), which, then, undergo increased glycolysis, similar to the anaerobic glycolysis or “Warburg” effect observed in cancer cells. This effect may contribute to the proliferative, apoptosis resistant, cancer-like phenotype observed in PASMCs, pulmonary artery endothelial cells, and adventitial fibroblasts in established PAH. Increased cytosolic calcium, probably due to changes in ion channels, also seems to contribute to the contractile, hyperproliferative, and anti-apoptotic phenotype of PAH PASMCs (24). It has also been suggested that an infection (e.g., viruses) from propagating a number of inflammatory pathways that lead to vascular cell proliferation, migration, and extracellular matrix deposition may contribute to the structural remodeling characteristic of PAH (32, 33). In this context, Kim et al. demonstrated that patients with PAH exhibit a unique gut microbiome profile that produces bacterial metabolites and molecules, which may play a role in the pathogenesis of PAH (34). The obstructive vascular remodeling increases right-ventricle (RV) afterload leading to its hypertrophy. Over time, changes in the RV such as fibrosis and ischemia may develop causing RV dysfunction [(24); Figure 1F].

It has also been suggested that portopulmonary hypertension (PoPH), which is a serious complication of chronic liver diseases that course with portal hypertension, may contribute to the pathogenesis of Sch-PAH (6, 18, 26). Portopulmonary hypertension pathogenesis is unknown, but several hypotheses have been proposed: (1) imbalance of vasoconstrictive and vasodilatory mediators is the most widely accepted explanation. According to it, mediators that are normally metabolized by the liver, reach the pulmonary circulation via portosystemic shunts causing PoPH; (2) hyperdynamic pulmonary circulation with increased RV output and blood flow through the pulmonary vascular bed causing increased sheer stress on the vascular wall and then PoPH; (3) genetic predisposition; (4) pulmonary thromboembolism from the portal venous system. Emboli from the portal circulation may reach pulmonary vessels through portosystemic shunts causing PH; and (5) inflammation (6, 35).

### Clinical Profile and Diagnosis

The clinical manifestations of Sch-PAH are indistinguishable from those of PAH of the other etiologies and result from progressive right-heart failure, including shortness of breath on exertion, peripheral edema, and syncope. However, pulmonary artery enlargement is more pronounced in Sch-PAH, independently of mean pulmonary artery pressure level, suggesting that this is more likely a feature of Sch-PAH (36). Furthermore, the clinical course of Sch-PAH seems to be more benign than that of idiopathic and connective tissue disease-associated PAH (37, 38).

As Sch-PAH is an important cause of PAH worldwide, particularly in developing countries, it is worth investigating this helminthiasis in people with PAH. The diagnosis of schistosomiasis is based on the history of environmental exposure to the infection, prior treatment of the parasitosis, or detection of eggs in stool, urine or rectal biopsy. Serologic testing is more helpful in the evaluation of patients from non-endemic areas because of the high rate of false positive results in persons who live in schistosome endemic areas (14, 17). Techniques to detect parasite antigens have been used and are commercially available in a point-of-care format (39). Polymerase chain reaction (PCR) and loop-mediated isothermal amplification (LAMP) on stool, urine or serum are sensitive but still of limited use. Hepatosplenic schistosomiasis diagnosis is based on ultrasonographic findings as previously described (23). Abdominal computed tomography (CT) scan and magnetic resonance imaging (MRI) may also demonstrate the findings of this form of schistosomiasis.

Echocardiography is usually the first exam performed in patients with suspicion of PH. Signs of RV overload such as RV hypertrophy and dilation, increase in pulmonary artery systolic pressure (PASP), and septal flattening or bulge are usually present. As measurement of pulmonary pressure by echocardiography is subject to limitations and its correlation with invasive measurements depends on optimal technical conditions, echocardiography is recommended for screening purposes. Studies have consistently shown that in nearly 50% of cases, PASP Doppler estimates differ by more than 10 mmHg from the values obtained by right-heart catheterization (40–42). However, echocardiogram can give important additional and prognostic information, such as right and left heart function, and right atrial area and presence of pericardial effusion, which are associated with prognosis (42, 43).

If PH probability is intermediate or high on echocardiography, it should be considered to perform a ventilation/perfusion (V/Q) scan, comprehensive heart evaluation, and pulmonary tests to rule out the WHO 2, 3, and 4 PH groups. Right-heart catheterization is mandatory to confirm the presence of PH and to distinguish pre-capillary and post-capillary predominance (22, 44).

### Clinical Management

Patients with Sch-PAH require close monitoring. Although reducing pulmonary pressure is the principal focus of the therapeutic approach, symptomatic, and supportive treatment remains an important component of patients' care. Diuretics are indicated to prevent or treat edema and the effects of volume overload on RV function and remodeling. Supplemental oxygen to maintain saturations above 90% helps to avoid hypoxia-induced pulmonary vascular constriction and consequent additional increase in pulmonary vascular resistance (4). Patients with portal hypertension have elevated bleeding risk from esophagogastric varices thus anticoagulation is usually not recommended (45).

Sch-PAH patients present improvements in functional class, 6-min walk distance, and hemodynamic parameters in response to PAH specific treatment (7, 46). Additionally, treated patients seem to have better survival rates than those untreated, at 5 years (47). Currently, PAH therapies target one or more of three major pathways implicated in disease progression: the nitric oxide (NO) pathway, which includes phosphodiesterase type 5 inhibitors (sildenafil and tadalafil) and soluble guanylyl cyclase stimulators (riociguat); the endothelin-1 (ET-1) pathway, which includes receptor antagonists (bosentan, ambrisentan, and macitentan); and the prostacyclin (PGI2) pathway, which includes prostacyclin analogs (epoprostenol, treprostinil, and iloprost) and the non-prostanoid IP-receptor agonist selexipag (7, 48). Considering resource limitations, only a few of these agents are available for clinical use in schistosomiasis endemic areas. Thus, the major drug used in those areas is sildenafil. However, as Sch-PAH shares pathophysiologic characteristics with the other WHO Group 1 PAH etiologies, creates the opportunity of treating Sch-PAH patients with the other agents (7). As a consequence of the better understanding of PAH pathophysiology, combination therapy, targeting the NO, ET-1, and PGI2 pathways, has emerged as the contemporary standard in the treatment of PAH patients (49). This approach requires investigation in Sch-PAH.

Sotatercept is an investigational ligand trap for TGF-β that was recently studied in patients with other forms of PAH. In these patients treated with standard therapy, the addition of sotatercept improved pulmonary vascular resistance, exercise tolerance, and serum levels of brain natriuretic peptide in comparison to placebo. As TGF-β activation may be a final common pathway in PAH pathogenesis, which is driven by different routes such as Th2 immune response in schistosomiasis, sotatercept may also be useful in the treatment of Sch-PAH patients (50).

The effect of anti-schistosome therapy in the lung parenchyma and vasculature has not been established. However, some authors suggest treating all Sch-PAH patients with praziquantel due to the severity of the disease and low risk of harm (51).

## Summary and Conclusions

Schistosome pulmonary arterial hypertension is one of the most common causes of WHO Group 1 PAH worldwide. Thus, even in non-endemic areas, epidemiologic information about schistosomiasis exposure is important to be elicited. This form of PH develops in patients with the severe hepatosplenic form of schistosomiasis, especially due to *S. mansoni* infection, following egg embolization to the lungs via collaterals that form as a consequence of portal hypertension. Although Sch-PAH pathogenesis is largely unknown, progressive remodeling of pulmonary vasculature is considered a key mechanism in the genesis of this disease. It has been suggested that TGF-β signaling as a consequence of the Th2 immune response elicited by eggs deposited into the lungs triggers the vascular remodeling. The clinical, laboratory, and hemodynamic profiles of Sch-PAH are similar to those of the other forms of PAH. Screening for the disease is performed using echocardiography, and definite diagnosis requires right-heart catheterization. Although patients with Sch-PAH show a more favorable hemodynamic profile and better survival compared to those with other forms PAH, its outcome remains poor, carrying a significant morbidity and mortality. Data on treatment of Sch-PAH patients with pulmonary vasodilators are limited, but recent evidence suggests that such therapies improve symptoms and may also improve survival.

## Author Contributions

TF, AA, and CF: conception and design of the article, literature review, drafted the article, revised it critically, and approved the submitted version. IG: drafted and prepared the final version of the figure, and approved the submitted version. All authors contributed to the article and approved the submitted version.

## Conflict of Interest

The authors declare that the research was conducted in the absence of any commercial or financial relationships that could be construed as a potential conflict of interest.

## Publisher's Note

All claims expressed in this article are solely those of the authors and do not necessarily represent those of their affiliated organizations, or those of the publisher, the editors and the reviewers. Any product that may be evaluated in this article, or claim that may be made by its manufacturer, is not guaranteed or endorsed by the publisher.
